# Barriers and facilitators to implementation of mental capacity legislation in care homes for older adults in the United Kingdom: a mixed-methods systematic review

**DOI:** 10.1093/ageing/afaf119

**Published:** 2025-05-15

**Authors:** Louis Stokes, Michelle Maden, Nefyn Williams, Nina Jacob, Sion Scott, Victoria Shepherd, Cara Gates, Liz Jones, Sandra Barker, Marie-Clare Hunter, Grahame Smith, Hayley Prout, Mishel Ingle, Ffion Curtis, Ruaraidh Hill, Alys Wyn Griffiths

**Affiliations:** The University of Sheffield Institute for Translational Neuroscience, The University of Sheffield, 358a Glossop Road Sheffield, Sheffield, South Yorkshire S10 2HQ, United Kingdom; Liverpool Reviews and Implementation Group Liverpool, University of Liverpool, Liverpool, United Kingdom; Institute of Population Health, University of Liverpool, Liverpool, United Kingdom; Centre for Trials Research Cardiff, Cardiff University, Cardiff, Wales, United Kingdom; School of Allied Health Professions, University of Leicester, University Road, Leicester, Leicestershire LE1 7RH, United Kingdom; Centre for Trials Research Cardiff, Cardiff University, Cardiff, Wales, United Kingdom; School of Health and Community Studies, Leeds Beckett University, Leeds, United Kingdom; The University of Sheffield Institute for Translational Neuroscience, The University of Sheffield, 358a Glossop Road Sheffield, Sheffield, South Yorkshire S10 2HQ, United Kingdom; The University of Sheffield Institute for Translational Neuroscience, The University of Sheffield, 358a Glossop Road Sheffield, Sheffield, South Yorkshire S10 2HQ, United Kingdom; The University of Sheffield Institute for Translational Neuroscience, The University of Sheffield, 358a Glossop Road Sheffield, Sheffield, South Yorkshire S10 2HQ, United Kingdom; School of Nursing and Allied Health, Liverpool John Moores University, Liverpool, United Kingdom; Centre for Trials Research Cardiff, Cardiff University, Cardiff, Wales, United Kingdom; The University of Sheffield Institute for Translational Neuroscience, The University of Sheffield, 358a Glossop Road Sheffield, Sheffield, South Yorkshire S10 2HQ, United Kingdom; Liverpool Reviews and Implementation Group Liverpool, University of Liverpool, Liverpool, United Kingdom; Liverpool Reviews and Implementation Group Liverpool, University of Liverpool, Liverpool, United Kingdom; The University of Sheffield Institute for Translational Neuroscience, The University of Sheffield, 358a Glossop Road Sheffield, Sheffield, South Yorkshire S10 2HQ, United Kingdom

**Keywords:** mental capacity, dementia, care homes, qualitative, social care, systematic review, older people

## Abstract

**Objective:**

Mental Capacity legislation defines when a person lacks capacity and subsequently supports individuals to make as many decisions as possible for themselves. Whilst frameworks exist, care home staff often feel unsupported with insufficient knowledge and training. This review aimed to understand barriers and facilitators of implementing mental capacity legislation in care homes for older adults in the United Kingdom.

**Methods:**

A systematic review was conducted and 3041 potentially relevant studies identified, with 13 studies eligible for inclusion. 11 focused on the Mental Capacity Act (2005) and two on the Adults with Incapacity (Scotland) Act 2000. Barriers and/or facilitators were extracted and subsequently mapped to the Capability, Opportunity and Motivation model and Theoretical Domains Framework.

**Results:**

Barriers included poor access to training, low staff confidence and a lack of understanding about using legislation in context. Conversely, staff reported in-person training using real-life examples, robust organisational policies and processes and respecting person-centred care were key facilitators. Sense-checking conversations were conducted with care home staff (n = 18) to interpret findings in the context of current practice.

**Conclusions:**

This review presents complex and multi-faceted barriers preventing the implementation of mental capacity legislation in care homes for older adults. Whilst care home staff have now started to appreciate the importance of such legislation, insufficient time, resources and an inability to track staff knowledge prevents effective implementation of the law. Future research should explore how staff are trained about legislation and identify best practices.

## Key points

- There are complex and multi-faceted barriers preventing the implementation of mental capacity legislation in care homes.
- Barriers include poor access to training, low staff confidence and poorly implemented organisational processes.
- Future research should further explore the implementation of legislation and develop best practice recommendations.

## Introduction

People with dementia experience progressive decline in cognitive abilities, including memory, reasoning and thinking [1]. As dementia progresses, most people lose capacity to make decisions for themselves [2]. This can fluctuate over time, be impacted by medication or concurrent illness and does not necessarily impact all decisions [3]. Almost one million people live with dementia in the UK, mainly aged over 65 years old, and many reside in care homes [4, 5].

The Mental Capacity Act 2005 (MCA) is a law in England and Wales designed to define when a person lacks capacity and support individuals to make as many decisions as possible [6]. Equivalent laws exist in Scotland (the Adults with Incapacity Act, 2000) and Northern Ireland (Mental Capacity Act [Northern Ireland], 2016(3)), respectively [7, 8]. Frameworks exist to guide care professionals to assess capacity and, in the case of diminished capacity, make best interest decisions. However, care home staff feel unsupported, with insufficient training to navigate these situations [9, 10]. Care home staff are not always aware of MCA principles, do not follow processes according to guidance and feel unable to support residents to make decisions [11].

Further research is needed to evaluate the effectiveness of MCA training and identify avenues for improvement [12]. Firstly, we must understand how legislation is implemented, and what barriers and facilitators exist. The role of behavioural science in understanding the influence of these in practice is widely recognised. The Capability Opportunity Motivation model (COM-B) proposes that behaviour is a system of interacting factors and successful behaviour change relies on the essential elements of capability, opportunity and motivation [13]. The Theoretical Domains Framework (TDF) provides a more detailed synthesis of behaviour change organised into 14 domains, e.g. ‘Knowledge’, ‘Beliefs about capabilities’ and ‘Social/professional role and Identity’ [14]. Both models have been extensively applied in evidence syntheses, to identify barriers and facilitators to behaviour change (COM-B) and understand behaviours (TDF). This systematic review aimed to identify and explore the barriers and facilitators of implementing the MCA, or equivalent capacity legislation, in care homes for older adults in all four nations.

## Methods

This review was conducted in accordance with Joanna Briggs Institute guidance and adhered to the Preferred Reporting Items for Systematic Reviews and Meta-Analyses (PRISMA) Guidelines [15, 16]. The review protocol was registered on PROSPERO (CRD42023444209).

### Search strategy and selection criteria

Following initial scoping searches, and search strategy refinement (see Supplementary File 1), the following databases were searched: MEDLINE, APA PsycINFO, Embase, CINAHL, Social Care Online, Social Policy and Practice, Health Management Information Consortium (HMIC), Scopus, Google Scholar, LENS.org and NIHR Journals Library. Forwards and backwards searching of included studies was conducted in November 2023. Key search terms included mental capacity legislation and care home setting terms. References of relevant systematic reviews were screened. Searches were also conducted through the National Grey Literature Collection and relevant authors contacted.

We focused on research exploring barriers to and/or facilitators of implementing mental capacity legislation in care homes, or training experiences, using any methodology (see Table 1). In the context of this systematic review, a barrier is a factor that hinders or impedes the implementation of legislation, and a facilitator is a factor that helps or enables the implementation of, or training about, mental capacity legislation in care settings with older adults. Barriers and facilitators could include physical determinants (e.g. availability of resources) or behavioural determinants (e.g. staff attitudes towards training).

**Table 1:** Inclusion and exclusion criteria for eligible studies.

**Inclusion criteria** Participants: Care home staff working with older adults (i.e. people aged 65 or over), residents or relatives. Study design: Qualitative, quantitative or mixed-methods research exploring barriers and facilitators to implementing mental capacity legislation. Setting: Care homes in England, Wales, Scotland or Northern Ireland. Date restrictions: Published after relevant legislative acts: Mental Capacity Act (England and Wales; 2005), Adults with Incapacity Act (Scotland; 2000) or Mental Capacity Act (Northern Ireland; 2016).
**Exclusion criteria** Study design: Secondary data analysis. Setting: Home care (domiciliary), secondary and tertiary hospital healthcare and the community, hospice settings, or those working exclusively with younger adults or children. Others: Studies not published in English or Welsh, or full-text unavailable.

### Study selection

Search results were imported into Endnote19 for deduplication, and then uploaded to Covidence for screening [17, 18]. Two reviewers screened titles and abstracts (LS, NJ/HP/MM), and full texts (LS, NJ) against the inclusion criteria. Eligibility conflicts were resolved through consultation with the wider research team (AWG/NW/SS). In addition to providing guidance on the research question, methodological approach and design, twenty included and excluded studies were also screened by the Lay Advisory Group, comprising seven public contributors who have direct experience of supporting older adults living with dementia. Two public contributors contributed to data synthesis and reporting of the systematic review (SB and MCH).

### Assessment of study quality

Quality assessment was independently conducted by two authors by applying the Critical Appraisal Skills Programme Qualitative Checklist [19]. All included studies adopted a qualitative methodology, or where present quantitative data were transformed to qualitative data to enable mapping to COM-B concepts and TDF domains. There were no quality criteria for inclusion.

### Data extraction and review synthesis

Data were extracted by two reviewers into a bespoke data extraction tool, and included study author, publication year, sample size, Act/legislation of interest, population, study design and phenomena of interest [20]. PROGRESS-Plus was used to map out the reporting of protected characteristics in the primary studies, and an Equality Impact Assessment (https://arc-em.nihr.ac.uk/arc-store-resources/equality-impact-assessment-eqia-toolkit) was completed to understand any health equality factors in relation to the review methods, findings and implications [21]. Additionally, author interpreted summaries and raw data pertaining to barriers and facilitators were extracted. The source of, and number of people endorsing, the barrier or facilitator, whether data were provider or patient reported, and the author-interpreted theme or broad categorisation were extracted where available.

Extracted barriers and facilitators data were analysed and synthesised by applying the principles of thematic synthesis [22]. Data were coded inductively, and themes developed. Data were deductively mapped to relevant COM-B and TDF domains by one author (LS) and checked for agreement (SS). Disagreements were discussed and consensus reached. To enable analysis and mapping, quantitative data were converted to qualitative data, or *qualitised* [15].

Following data synthesis, ‘sense-checking’ was conducted with older adult care home staff, through either one-to-one conversations or focus groups. The review methodology and an overview of the included studies, detailing key barriers and facilitators, were presented to staff. Participants provided insight into whether findings resonated with their experiences.

## Results

**Figure 1:**
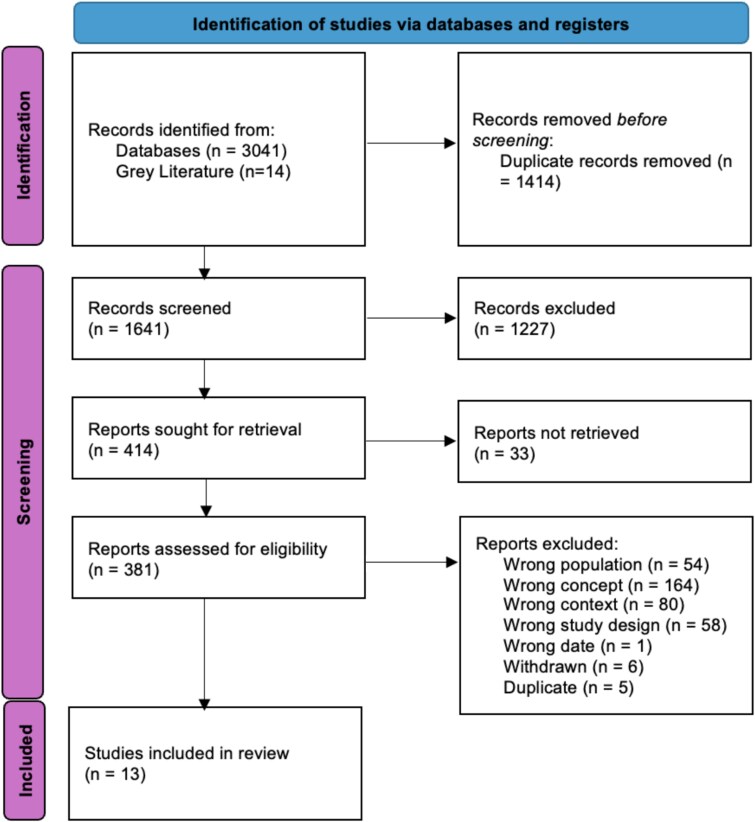
PRISMA flow diagram of screening process.

We identified 3014 potentially relevant studies and, removed 1414 duplicates. Titles and abstracts of 1641 studies were screened, of which 381 were screened at full-text, and 13 were deemed eligible for inclusion (see Figure 1). No additional studies were identified following backward searching. Included studies are summarised in Table 2.

**Table 2:** Characteristics of included studies.

**Author(s), country, year of publication**	**Sample size (n)**	**Act/legislation of interest**	**Population**	**Study design**	**Phenomena of interest**
Burns & Watson, UK, 2009 [23]	11	Adults with Incapacity (Scotland) Act (2000)	Care home managers (11)	Face-to-face and telephone interviews	Assess the effectiveness of the AWI Part 4 and experiences of using the legislation
Davidson, Wilkinson, Urquhart et al., UK, 2004 [24]	3^a^	Adults with Incapacity (Scotland) Act (2000)	Care home manager (1), registered nurse (2)	One-to-one semi-structured interviews	Exploring the awareness and experiences of social care staff using the AWI Act in practice
Fetherstone, Hughes & Woods, UK, 2022 [25]	10	MCA (2005)	Care home staff (10)	One-to-one semi-structured interviews	Explore thought processes underlying staff decision making and staff knowledge of the MCA principles
Gough & Kerlin, UK, 2012 [26]	9^a^	MCA (2005)	Care home deputy managers and managers (9)	Focus group	Evaluate the impact of MCA training within older persons’ care homes
Jayes, Austin, & Brown, UK, 2022 [27]	29	MCA (2005)	Care home managers (18), registered nurses (17), care assistants (4)	Five semi-structured focus groups	Understand the current challenges faced by care home staff when supporting residents to make decisions and explore staff members’ needs in the context of the MCA
Kuylen, Wyllie, Bhatt et al., UK, 2022 [28]	138^a^	MCA (2005)	Professions working in or with care homes	Survey responses (120) and focus groups (18)	Experiences of professionals who worked with care home residents with impaired capacity in England and Wales during the COVID-19 pandemic
Manthorpe, Samsi, Rapaport et al., UK, 2012 [29]	45^a^	MCA (2005)	Care home managers, senior care staff and care home workers	One-to-one semi-structured interviews	Explore the reflections of dementia care professionals with family experiences of dementia on decision-making frameworks
Manthorpe & Samsi, UK, 2015 [30]	20^a^	MCA (2005)	Care home staff	One-to-one semi-structured interviews	Exploration of how the MCA is implemented in community-based dementia care
Manthorpe & Samsi, UK, 2013 [31]	272	MCA (2005)	Community social care staff	One-to-one semi-structured interviews	Understand the experiences of social care staff applying the MCA
Manthorpe, Samsi, Heath et al., UK, 2011 [32]	32	MCA (2005)	Senior care staff and care workers	One-to-one semi-structured interviews	Identify staff challenges when using the MCA in practice with people living with dementia and the expectations of care staff about the MCA
Manthorpe & Samsi, UK, 2016 [33]	27	MCA (2005)	Care home staff	One-to-one semi-structured interviews	Investigate the implementation and adoption of the MCA in dementia practice
Stewart, Goddard, Schiff et al., UK, 2011 [34]	90^a^	MCA (2005)	Care home staff	One-to-one semi-structured interviews	Explore the views of care home staff about advance care planning in care homes for older people
Williams, Boyle, Jepson et al., UK, 2013 [35]	9^a^	MCA (2005)	Social care professionals	One-to-one interviews (9)	Understand whether the MCA and available guidance are relevant and sufficient for the different situations encountered in health, social care and legal contexts

^a^where data is available, the study sample size has been adjusted to reflect the total number of care home staff participating in the study and subsequently included in this review

### Study characteristics

Twelve qualitative and one mixed-methods studies published between 2004–2022 were included. Studies included care home managers and/or deputy managers [23, 24, 26–29], registered nurses [24, 27, 28, 31, 35] and care home staff [25, 27–35]. Qualitative studies involved interviews (*n = 519*) [23–25, 29–35] and focus groups (*n = 38*) [26, 27]. One mixed-methods study included questionnaires (*n = 120*) and focus groups (*n = 18*) [28]. Eleven studies focussed on the MCA (2005) [25–35] and two studies on the Adults with Incapacity (Scotland) Act 2000 [23, 24]. No studies explored implementation of the Mental Capacity Act (Northern Ireland; 2016).

Our Equality Impact Assessment identified that the review had broad inclusion criteria (accounting for target group, e.g. care homes for older adults) alongside a comprehensive search (developed by an experienced information specialist), therefore it seems unlikely that the methodological approach would introduce or perpetuate existing health inequalities. Consultations with diverse stakeholder groups including public contributors, and care home staff engaged in the sense-checking workshops, ensured a broad range of opinions and perspectives were considered in the design, conduct and reporting of this review. The findings of this review could have a positive impact across many protected characteristics for care home residents in the future. That said, the assessment was partially limited by poor reporting in the primary studies (PROGRESS Plus) of protected characteristics (e.g. study locations, sex, ethnicity) which may have implications in terms of generalisability and implementation.

### Quality assessment

Twelve studies were rated as high quality (see Table 3).

**Table 3:** Quality assessment of included studies.

	Burns & Watson (2009)	Davidson et al. (2004)	Fetherstone et al. (2022)	Gough & Kerlin (2012)	Jayes et al. (2022)	Kuylen et al. (2022)	Manthorpe et al. (2012)	Manthorpe & Samsi (2015)	Manthorpe & Samsi (2013)	Manthorpe et al. (2011)	Manthorpe & Samsi (2016)	Stewart et al. (2011)	Williams et al. (2013)
Was there a clear statement of the aims of the research?	Yes	Yes	Yes	Yes	Yes	Yes	Yes	Yes	Yes	Yes	Yes	Yes	Yes
Is a qualitative methodology appropriate?	Yes	Yes	Yes	Yes	Yes	Yes	Yes	Yes	Yes	Yes	Yes	Yes	Yes
Was the research design appropriate to address the aims of the research?	Yes	Yes	Yes	Yes	Yes	Yes	Yes	Yes	Yes	Yes	Yes	Yes	Yes
Was the recruitment strategy appropriate to the aims of the research?	Yes	Yes	Yes	Yes	Yes	Yes	Yes	Yes	Yes	Yes	Yes	Yes	Yes
Were the data collected in a way that addressed the research issue?	Yes	Yes	Yes	Yes	Yes	Yes	Yes	Yes	Yes	Yes	Yes	Yes	Yes
Has the relationship between researcher and participants been adequately considered?	No	No	Yes	No	Yes	Yes	No	No	No	No	No	Yes	No
Have ethical issues been taken into consideration?	No	Yes	Yes	Yes	Yes	Yes	Yes	Yes	Yes	Yes	Yes	Yes	Yes
Was the data analysis sufficiently rigorous?	Can’t tell	Yes	Yes	Yes	Yes	Yes	Yes	Yes	Yes	Yes	Yes	Yes	Yes
Is there a clear statement of findings?	Yes	Yes	Yes	Yes	Yes	Yes	Yes	Yes	Yes	Yes	Yes	Yes	Yes
Is the research valuable?	Yes	Yes	Yes	Yes	Yes	Yes	Yes	Yes	Yes	Yes	Yes	Yes	Yes
Level of quality?	Medium	High	High	High	High	High	High	High	High	High	High	High	High

### Barriers and facilitators

The barriers and facilitators identified in the included papers were relevant to all COM-B and 11 of the 14 TDF domains (*Knowledge; Memory, attention and decision processes; Skills; Environmental context and resources; Social influences; Social/professional role and identity; Belief about capabilities; Goals; Optimism.* No barriers or facilitators were mapped to *Behavioural Regulation, Intentions and Emotion.* See Table 4 for examples of mapped data.

**Table 4:** Examples of extracted raw data mapped to TDF.

Interpretation (barrier/facilitator) linked to TDF domains	Examples of extracted data
*Barriers*	
Belief from care home staff that MCA legislation had been eased during COVID-19 pandemic [*Knowledge*]	*‘I have had a number of care homes telling me that there were easements to the MCA, when there never were.’* [28]
Lack of training provision available to care home staff regarding use of mental capacity legislation [*Environmental context and resources]*	*‘There is not enough training on it. We need more training.’* [32]
Deferring implementation of MCA to other staff, attributed to qualifications [*Social/professional role and identity*]	*‘they’ve got that nursing degree haven’t they, so they can make that decision and it will be a legal decision, whereas mine wouldn’t be’* [27]
Staff practicing forced care, rather than person-centred care with supported decision-making [*Skills*]	*“Talk to them about their interests and take their mind off the task that you are doing so you do what you need to do really."* [25]
Documentation did not demonstrate MCA workload or decision-making, not capturing important meetings or conversations [*Environmental context and resources*]	*‘it’s all them other bits of conversations in between (meetings) that are not shown through on that document there’* [27]
Staff considered other professionals' practice poor, inconsistent with legal standards [*Social influences*]	*‘when you go to do a preadmission assessment, the actual capacity assessment that’s run in the hospital is either non-existent or very minimal’* [27]
*Facilitators*	
Ability for staff to interpret physical cues to communicate with residents who have communication challenges [*Skills]*	‘*Some people can’t verbalise, but they can communicate, but by other means, actually by facial expressions or with their body language’* [27]
Increased confidence of care home staff to embed MCA in daily practice [*Knowledge]*	*"We do have to explain that [the MCA] but we are doing it with more confidence now – it comes more easily, so it’s not so much of a disadvantage now"* [31]
Staff personal experiences of navigating MCA provided context when implementing in professional practice *[Knowledge*]	*"Own experiences? This morning I’ve spent hours dealing with family. . . I’m right in the middle of it. . . an emphatic ‘yes’ and mental capacity is all part of it. The MCA has affected both myself and my partner. . . it’s been an enormous help. . . Yes, I know how it is to be a carer.”* [29]
Staff belief in important of delivering person-centred care [*Social influence*]	*‘It’s about involvement. . .so that they feel like a person and that they’re valued’* [27]
Best practice in implementation of legislation embedded across all staff in the care home [*Social influence*]	*“all the staff get involved in that, carers, housekeepers, nurses, everybody who’s working in the home"* [27]
Staff viewed MCA as protective against questions or doubts about own practice and abilities [*Reinforcement*]	*"To professionals it’s like insurance, isn’t it? Like a form of protection; and you’re an enabler rather than a controller. To carers? I think it serves to alleviate their anxiety by reassuring them that relatives have that choice"* [31]

### Barriers

#### Capability-related barriers

Care home staff possessed insufficient knowledge about the existence, and purpose, of mental capacity legislation [24–26, 28, 30, 32, 33, 35]. Staff had less knowledge than other health and social care professionals, e.g. social workers, and often did not understand that capacity was decision-specific [28, 30, 35]. Whilst staff possessed a basic understanding of relevant legislation, applying this in practice remained challenging [26, 30, 32, 33]. Misconceptions included that by virtue of a dementia diagnosis residents were unable to make any decisions, and had impaired capacity, or the blanket belief that legislation did not apply to care homes [25, 30, 32, 33]. Some staff lacked understanding about practical implementation in the context of person-centred care and subsequently practised forced care [25]. Staff described an inability, or lack of skills, to interpret and practically implement the legislation and supporting documentation [23, 27]. They also reported insufficient knowledge, and skills, to support shared decision-making through meaningful communication with residents with differing needs [27].

#### Opportunity-related barriers

Care home staff attributed communication challenges to poor access to speech and language therapists or translation services, where residents used English as an additional language or when they did not communicate verbally [27, 28, 33]. Poor knowledge of legislation was linked to variable access to training, insufficient awareness of guidance to support implementation and detail of how to practically embed recommended processes [23, 31–33]. Some staff perceived that MCA legislation was not suitable to the current context, using archaic terminology [23]. Particular concern was cited regarding the applicability and relevance during the COVID-19 pandemic [28]. Staff felt that understanding and implementing MCA legislation was time-consuming, and therefore often deprioritised [27, 31, 33]. Insufficient time was a barrier to reading relevant legislation literature, to be able to implement this effectively [32]. Care home managers reported funding, and arranging cover to facilitate staff training, as a barrier. This was exacerbated in smaller care organisations due to smaller budgets and staff teams [23, 24, 26, 28]. Less robust processes in single care homes, or smaller organisations, also contributed to poor availability of documentation to support implementation of legislation and joint decision-making, and staff were unable to produce legally compliant documentation to satisfy regulatory inspections [23, 27]. Care home staff who had accessed training felt aspects pertinent to mental capacity legislation were often delivered in silo, rather than as an integrated model to aid practical implementation [26, 30].

The need to educate family members about mental capacity legislation, challenge misconceptions and navigate disagreements about a person’s best interests presented an additional barrier to implementation in practice [27, 29, 32, 34]. Staff experienced challenges regarding their own views about applying a best interests decision when it conflicted with their role-specific training regarding resident autonomy [35]. Conflict also emerged when staff perceived that other professionals did not follow best practices, including not seeking the views of residents in decision-making, making incorrect best interest decisions due to poor knowledge of residents, and insufficient capacity assessments during transfers between care settings [27].

#### Motivation-related barriers

Managers felt they lacked expertise to promote compliant processes and practices, and were stressed about being primarily responsible for making decisions—particularly in relation to financial decisions under the Adults with Incapacity (Scotland) Act (2000) [23, 31, 32]. Staff confidence in applying legislation was low [23, 27, 31–33, 35], and they relied on external agencies or better qualified staff to facilitate more complex decisions, including conducting capacity assessments and implementing outcomes [27]. Alongside low confidence, some staff did not consider that understanding and implementing mental capacity legislation was within scope of their role, or experienced difficulties with concepts such as advance care planning owing to differing cultural beliefs and worries about making decisions reserved for family members [34]. Subsequently, staff often showed minimal interest in learning about mental capacity legislation to improve the delivery of person-centred care [25, 32, 33]. During the COVID-19 pandemic, some staff considered that blanket approaches to restrictions conflicted with the principles of the MCA and found these challenging to enact [28].

### Facilitators

#### Capability-related facilitators

The most frequently cited facilitator to successful implementation was staff valuing the rights of resident autonomy in line with legal requirements, and acknowledging how the underlying principles of mental capacity legislation protected these rights [27, 34, 35]. Practical implementation relied on staff understanding of adapting communication styles to support residents to demonstrate their decision-making abilities, for instance by pointing to different options when offering choice, identifying non-verbal cues such as body language, or using visual communication aids [27]. Breaking decisions down into stepped choices for residents, developing personal knowledge of the individual being cared for, and gaining insights of preferences from family members also supported decision-making [31]. Strong working knowledge of mental capacity legislation and appreciation of this in the context of dementia was imperative for care home managers [33–35].

#### Opportunity-related facilitators

Organisational processes supported the identification of decision-specific capacity changes, and clear policies guided implementing legislation in practice [23, 27, 33]. In-house training reduced staffing challenges and was more cost-effective from the perspective of care home managers [26]. Staff valued bitesize training delivered at convenient times, such as at handover, incorporating real-life case scenarios, role play, ongoing supervision and regular training updates [26, 31]. The development and routine use of mental capacity documentation supported staff to follow processes consistently, and staff valued the utility of checklists to justify decisions [26, 33]. Best practice sharing opportunities, including team meetings or huddles, and working jointly with colleagues, were valuable opportunities for staff to develop knowledge of legislation outside of training sessions [25, 33]. This allowed staff to resolve problems relating to practical implementation [25, 33]. Workplace culture contributed to successful implementation, with an emphasis on person-centred care, forming and nurturing working relationships with residents, and valuing dignity, humanity and respect [25, 27, 31–35]. Beyond the presence of a positive workplace culture, perceived alignment between the MCA principles and organisational processes supporting person-centred care was important [31, 34, 35].

#### Motivation-related facilitators

Legislation provided care home staff with confidence, offering protection to defend contentious decisions, allegations, or challenges against their practice. Staff noted the national press had featured high-profile cases involving the utility of legislation in ill-treatment cases [27, 30, 31, 33]. Some staff considered that legislation also offered protection to residents who lacked capacity [35]. Incorporating person-centred care and routine support for autonomous decision-making into the professional carers’ identity facilitated the routine, consistent and meaningful inclusion of mental capacity legislation into daily practice, increasing confidence in implementation [27, 31, 32]. Managers who possessed a strong understanding of mental capacity legislation felt able to take leadership regarding implementation, and subsequently supported staff to discuss case scenarios or challenging situations in a more open organisational culture [26, 27, 35].

### Sense-checking conversations

To consider the relevance of findings to current practice, 18 care home staff participated in sense-checking conversations through one-to-one interviews (n = 9) and focus groups (n = 3 and n = 6). (see Table 5 for demographics).

**Table 5:** Demographics of participants included in sense-checking workshops (n = 18).

Participant demographics	
Age	Under 16 (0), 16–19 (0), 20–29 (5), 30–44 (11), 45–59 (1), 60–74 (1), 75 and over (0)
Gender	Female (13), Male (4), Non-binary (1)
Sexual orientation	Bisexual (2), Gay or lesbian (2), Heterosexual (12), Prefer not to say (2)
Ethnic group	Black or Black British African (2), Black or Black British Caribbean (2), White British (12), White Welsh (1), Other White background (1)
Occupation	Administrator (2), Management (6), Housekeeping (2), Healthcare assistant (8)
Time in role	<1 year (6), 2–5 years (5), 5–10 years (4), Longer than 10 years (3)

Participants unanimously agreed that mental capacity legislation was challenging to understand and implement in daily practice. Unlike literature identified in this review, all participants felt that following a dementia diagnosis it was important to support people to make decisions and respect their autonomy. However, staff shared examples of practice that conflicted with this, including a lack of decision specific capacity assessments, and blanket monthly reviews of capacity.

All participants received online training about the MCA. Participants felt that this provided them with a foundational knowledge of the core underpinnings of mental capacity legislation, but found it challenging to take this knowledge and subsequently implement learnings within their daily practice. Participants felt that in-person group-based training would enable staff to share real-life examples of decision-making and empower staff to put knowledge into practice.

Areas of difficulty included managing family conflict around decision-making, explaining the role of legislation when the resident had capacity to make their own decisions, and supporting residents to make unwise decisions. Related to unwise decisions, staff worried they could be liable should any harm come to the resident, and actively risk assessed situations.

Whilst most staff were aware of where to find appropriate policies and processes, few felt that they would proactively seek these and read them. Conflicting with reports independent care homes may have fewer processes in place, smaller care homes felt that they could be more reactive and flexible with their processes than larger organisations.

Overall, participants felt staff could be better supported around understanding and implementing mental capacity legislation. Whilst many staff did not resonate with the principles or terminology of legislation, it was easy to identify how and where they applied this. This demonstrates the importance of using lay language and real-life examples when translating complex legislation into daily practice in care homes.

## Discussion

### Summary of main findings

The review highlights multi-faceted barriers and facilitators to implementation of mental capacity legislation in care homes for older adults in the United Kingdom. Knowledge misconceptions about capacity following a dementia diagnosis and challenges translating guidance into practice were notable capability-related barriers. Opportunity-related barriers included variable access to, and poor awareness of, training, supporting and managing family relationships relating to decision-making and personal conflict when applying best interest decisions. Low confidence to interpret and apply legislation, not understanding the remit of legislation in relation to role-related responsibilities and a lack of interest in learning about such legislation were motivation-related barriers. Facilitators included practical support to overcome communication challenges with residents and support to maintain a working knowledge of legislation in the context of dementia. Important factors for successful implementation included robust organisational processes to identify capacity changes, and clear policies embedding guidance into practice, alongside accessible training incorporating real-life scenarios. A positive workplace culture encouraging best practice sharing and collaborative problem solving was also important. Motivation-related facilitators included perceptions that legislation provided protection against allegations or challenges against staff practice, and consistent and routine support from management for resident-led decision making irrespective of capacity. No data was mapped to 3 of the 10 TDF domains, which is unsurprising given that none of the included studies were underpinned by the framework. However, future research should explore the links between implementation of mental capacity legislation with staff motivation, particularly the impact of intentions, emotions and reinforcement.

### Comparison with previous literature

Core barriers to the implementation of mental capacity legislation included knowledge, training and understanding how to embed guidance into practice [36]. This aligns with staff supporting people with learning disabilities also demonstrating a highly variable understanding of the MCA and related decision-making [37]. Care home staff often relied on the expertise of senior colleagues when making decisions [33]. This is particularly problematic as managers often felt under pressure and under qualified to be the primary decision maker, and that less qualified staff are unlikely to challenge decisions made by senior staff [38].

Poor knowledge of legislation was frequently attributed to a lack of, or inaccessible, training. Staff wanted to access shorter training at convenient times, relatable to real-life experiences and delivered through a top-down approach by the organisation. Whilst our recent scoping review identified that a one-size-fits-all approach to MCA fails to account for the differing needs of stakeholders; limited consensus was reached regarding best practices for the method of training—although staff favoured face-to-face training incorporating practical learnings [39]. Previous research has concluded that improved training strategies, in line with the unique needs of individual staff members, were necessary to translate the principles underlying MCA into practice and that current training was too infrequent and theory-focused [40, 41]. Within this review, managers did not feel that they could track staff training or adequately identify knowledge gaps. Training outcomes are measured and tracked ineffectively in other social care environments [40], meaning that very little is known about current training outcomes. This is critical to identify knowledge gaps and support staff to develop well-rounded understanding, further supported by ongoing supervision and translating knowledge to practice in a pragmatic and meaningful way [42].

In this review, care home staff described a moral dilemma between implementing best interest decisions and respecting resident autonomy. Mental capacity legislation was seen as protective for both staff and residents, supporting the ability of care staff to balance these two perspectives when making decisions. Within other sectors, staff decisions are shaped by personal life experience and values—a clear discrepancy with the approach required by legal frameworks [43]. Many care staff had personal/familial experience of dementia, which influenced their practice and often influenced career decisions [32]. Reliance on personal experience is a strategy implemented by care staff, possibly based on their fear of making incorrect decisions. The value of nurturing and supporting staff to actively take risks, and designing services around this concept, were emphasised as important changes to current infrastructure [44, 45].

Whilst the findings indicate increased acceptance of mental capacity legislation over time, and acknowledgement of it as a protective mechanism rather than as burdensome paperwork unlikely to change practice, gaps in knowledge and misconceptions are still evident. Staff remain unsupported to effectively learn about, and subsequently use, legislation in a meaningful and practical way. Implementation could be improved by learning from other legislation, including England’s Care Act 2014, and policymakers engaging reflectively with legislation over time to meet the needs of a changing workforce and context [46].

### Strengths and limitations

This is the first systematic review to apply behaviour change models to understand key barriers and facilitators of implementing mental capacity legislation in older adult care homes. Selection bias and potential error were reduced by rigorous screening and quality assessment. The inclusion criteria meant a range of viewpoints were incorporated, enabling diverse perspectives. The collection of PROGRESS-plus data assessed protected characteristics relevant to health equity. Sense checking our findings with care home staff ensured they were appropriate, relevant and contextualised. Given the focus on legislation in the four UK nations, findings may not be applicable in other jurisdictions where different legal frameworks are in place, or do not exist. Only primary data relating to the care of older adults were included, and there may be relevant learnings from other health and social care settings. Additionally, despite proposing a mixed-methods systematic review only data qualitatively describing barriers and facilitators were available, should the review be updated in the future, with further research conducted it is hoped that both qualitative and quantitative studies could be synthesised.

### Implications for practice and future research

Whilst care home staff appreciate the importance of mental capacity legislation, insufficient time, resources and an inability to track whether staff possess adequate knowledge prevents effective implementation [40]. Training must meet the needs of staff, including examples of how to embed learnings in practice, and weave guidance into all aspects of care. Staff have diverse backgrounds, experiences and motivations for working in social care. Valuing the personal and professional expertise of care staff, and the knowledge they develop about residents over time, is crucial to delivering meaningful person-centred care. Future research should identify best practice in training from a range of care organisations varying in size, degree of socio-economic deprivation and structure. It is critical that protected characteristics seldom reported in the research identified in this review (study locations, sex, ethnicity) are collected and reported to ensure generalisability and implementation. This will ensure that future best practice recommendations regarding implementation of legislation are widely applicable.

### Conclusion

Although mental capacity legislation is crucial to meet the needs of care home residents, this review presents complex and multi-faceted barriers preventing the practical implementation of relevant guidance. Poor access to training, low confidence, and a lack of understanding about what mental capacity legislation means in practice are just some of the challenges presented. Poor understanding of what best practice training looks like in care homes, and disagreement about how to train staff effectively exists. Future research should seek to understand this and involve stakeholders in the development of best practice recommendations when developing, delivering and implementing training about mental capacity legislation in care homes.

## Supplementary Material

aa-24-2790-File002_afaf119
